# Effects of PPARγ and RBP4 Gene Variants on Metabolic Syndrome in HIV-Infected Patients with Anti-Retroviral Therapy

**DOI:** 10.1371/journal.pone.0049102

**Published:** 2012-11-07

**Authors:** Yuan-Pin Hung, Nan-Yao Lee, Sheng-Hsiang Lin, Ho-Ching Chang, Chi-Jung Wu, Chia-Ming Chang, Po-Lin Chen, Hsiao-Ju Lin, Yi-Hui Wu, Pei-Jane Tsai, Yau-Sheng Tsai, Wen-Chien Ko

**Affiliations:** 1 Institute of Clinical Medicine, National Cheng Kung University, Tainan, Taiwan; 2 Department of Internal Medicine, Tainan Hospital, Department of Health, Executive Yuan, Tainan, Taiwan; 3 Department of Internal Medicine, National Cheng Kung University Hospital, Tainan, Taiwan; 4 Center for Infection Control, National Cheng Kung University Hospital, Tainan, Taiwan; 5 Department of Nutrition, Chi-Mei Hospital, Tainan, Taiwan; 6 Division of Clinical Research, National Health Research Institutes, Tainan, Taiwan; 7 Department of Internal Medicine, Pingtung Christian Hospital, Pingtung, Taiwan; 8 Department of Medical Laboratory Science and Biotechnology, National Cheng Kung University, Tainan, Taiwan; 9 Research Center of Infectious Disease and Signaling, National Cheng Kung University, Tainan, Taiwan; 10 Department of Medicine, National Cheng Kung University, Tainan, Taiwan; Fundação Oswaldo Cruz, Brazil

## Abstract

**Background:**

PPARγ and RBP4 are known to regulate lipid and glucose metabolism and insulin resistance. The influences of PPARγ (C1431T and Pro12Ala) and RBP4 (−803GA) polymorphisms on metabolic syndrome in HIV-infected patients receiving anti-retroviral therapy were examined in this study.

**Materials and Methods:**

A cross-sectional study of HIV-1 infected adults with antiretroviral therapy for more than one year in the National Cheng Kung University Hospital was conducted. The gene polymorphisms were determined by quantitative PCR.

**Results:**

Ninety-one patients were included in the study. Eighty-two (90.1%) patients were males with a mean age of 44.4 years. For the C1431T polymorphism in PPARγ, while patients with the T allele (48.4%) had trends toward lower rate of hypertriglyceridemia, the borderline significance together with insignificant power did not support the protective effect of the T allele against development of hypertriglyceridemia. For the Pro12Ala polymorphism in PPARγ, although patients with the *Pro/Ala* genotype (8.8%) had a higher level of serum LDL (138.0 *vs*. 111.5 mg/dl, *P* = 0.04) and trends toward higher rates of hypercholesterolemia and serum LDL>110 mg/dl, these variables were found to be independent of the *Pro/Ala* genotype in the multivariate analysis. For the −803GA polymorphism in RBP4, patients with the A allele (23.1%) more often had insulin resistance (HOMA>3.8; 33.3 *vs*. 8.7%, *P* = 0.01) and more often received anti-hypoglycemic drugs (14.3 *vs*. 1.4%, *P* = 0.04). The detrimental effect of the A allele in RBP4 −803GA polymorphism on development of insulin resistance was supported by the multivariate analysis adjusting for covariates.

**Conclusion:**

The impacts of PPARγ C1431T and Pro12Ala polymorphisms on metabolism in HIV-infected patients are not significant. RBP4 −803GA polymorphism has increased risk of insulin resistance in HIV-infected patients with anti-retroviral therapy.

## Introduction

The annual, newly reported cases with human immunodeficiency virus (HIV) infection in Taiwan has been increasing since 2003, with an 11% increase in 2003, a 77% increase in 2004 and 123% increase in 2005 [1]. The introduction of highly active antiretroviral therapy (HAART) has dramatically decreased the morbidity and mortality in HIV-infected patients [2], [3]. However, prolonged use of HAART is associated with many metabolic complications, such as lipodystrophy, dyslipidemia and glucose metabolism disorder [4], [5]. The prevalence of metabolic syndrome among HIV-infected persons is 26% in the United States, and 81% met at least one of the criteria for the risk of metabolic syndrome regardless of the use of HAART or the type of HAART [6], [7]. A similar clinical issue was noted among HIV-infected patients receiving HAART in Taiwan. A study of 242 Taiwanese receiving HAART reported that 79 (32.6%) had hypertriglyceridemia (>250 mg/dL) [8]. In another study consisting of 877 HIV-infected Taiwanese, 210 (26.2%) had metabolic syndrome, especially in those with HAART [9]. Thus, metabolic complications are an important issue in HIV-infected patients under anti-retroviral therapy. However, not all HIV-infected patients receiving HAART develop metabolic syndrome. Many factors have been shown to contribute to these metabolic alternations, including genetics, cytokines, diet, drinking, gender and age [10].

Peroxisome proliferator-activated receptor γ (PPARγ), a nuclear receptor, stimulates lipid uptake and adipogenesis by fat cells and is a key regulator of lipid metabolism, adipogenesis and insulin resistance [11]. The actions of PPARγ are mediated by two protein isoforms, the widely expressed PPARγ1 and adipose tissue-restricted PPARγ2. Yen *et al.* performed a molecular scanning of human PPARγ in diabetic Caucasians and identified two variants in the coding region of the gene: Pro12Ala missense (rs1801282) and C1431T silent mutations (rs3856806; also known as His477His or C161T) [12]. Further studies identified that these variants are associated with insulin sensitivity and glucose metabolism in non-HIV population [13], [14]. An alanine substitution at position 12, located within the extra N-terminal residues of adipose tissue-restricted PPAR*γ*2, leads to a lower DNA-binding affinity and decreased transactivation in *in vitro* studies [13], [15]. The C1431T polymorphism is a silent (C>T) substitution at a nucleotide 1431 in exon 6. While the mechanism by which the C1431T mutation in PPARγ affects its activity remains unclear, it has been suggested that it is in linkage disequilibrium with mutations in other regions of the gene that regulate the activity of PPARγ [16]. The estimated frequencies of PPARγ Pro12Ala and C1431T polymorphisms in Chinese are about 7.2% and 44.3% [17].

Retinol-binding protein 4 (RBP4) is an adipocytokine, secreted from adipocytes and released into circulation [18]. Serum RBP4 levels are elevated in insulin-resistant mice and humans with obesity and type 2 diabetes, and are normalized by rosiglitazone, a PPARγ agonist [19]. Experiments in mice indicated that elevated RBP4 levels cause insulin resistance, suggesting that the improvement of glucose metabolism by PPARγ activation may be through the reduction of circulating RBP4 levels [20]. Furthermore, RBP4 −803GA polymorphism (rs3758539), located at 5′ upstream of the translational start site within a putative enhancer region, is associated with an increased risk of type 2 diabetes in non HIV-infected patients [21]. *In vitro* studies showed that −803 A allele induces greater transcriptional activity and higher binding affinity with the transcription factor hepatocyte nuclear factor 1 alpha (HNF1α) than the G allele, which may cause increased serum RBP4 levels in diabetic patients [22], [23]. Antiretroviral therapy in HIV-infected patients has been reported to induce a pronounced increase of plasma RBP4 [18], which is associated with obesity, insulin resistance and dyslipidemia [24]. However, the relationship between PPARγ or RBP4 polymorphism and insulin resistance or dyslipidemia in HIV-infected patients receiving HAART remains unclear. The objective of the present study is to investigate the effect of PPARγ and RBP4 polymorphisms on body mass, insulin resistance and dyslipidemia among HIV-infected patients with anti-retroviral therapy after adjusting other risk factors, including influence of anti-retroviral regimen, diet and drinking.

## Materials and Methods

A cross-sectional study of HIV-1 infected patients in the National Cheng Kong University Hospital was conducted. HIV-infected patients aged 18 years or older with regular outpatient follow-ups and antiretroviral therapy (efavirenz or lopinavir/ritonavir plus two nucleoside reverse transcriptase inhibitors [NRTIs]) for at least one year were included. Patients, who had diabetes mellitus (DM), dyslipidemia or lipodystrophy before the clinical diagnosis of HIV infection, or had active opportunistic infections, were excluded.

Demographic data, duration of HIV disease, regimen and duration of HAART were collected. Body height, body weight, waist and hip circumference, and blood pressure were measured at outpatient visits. The waist circumference was measured at the midpoint of the rib margin and the iliac crest at the end of expiration, and the hip circumference was measured at the greatest protuberance of the buttocks [25]. Central obesity was defined as waist >90 cm for men and >80 cm for women according to the Bureau of Health Promotion, Department of Health, Taiwan. The diet condition and habitual of the patients were assessed by a modified questionnaire [26], [27]. The nutrition content of the diet was analyzed. Daily metabolic rate and total energy consumption in calories (including resting energy expenditure and total energy expenditure [TEE]) were calculated from age, gender, weight and height of the patients [26]. Patients who ate more than TEE were regarded as intake over-TEE, otherwise under-TEE.

Overnight fasting blood samples were taken for the measurement of serum concentrations of glucose, insulin, triglyceride, low-density lipoprotein (LDL), high-density lipoprotein (HDL), and total cholesterol. Dyslipidemia was defined as either of serum cholesterol >200 mg/dl, triglyceride >150 mg/dl, LDL>110 mg/dl, or HDL<40 mg/dl for men and <50 mg/dl for women [28], [29]. Insulin resistance was estimated by the model of homeostasis model assessment (HOMA) [5], [17], which was derived from fasting insulin (µU/ml) × fasting glucose (mmol/l)/22.5. A HOMA score higher than 3.8 was regarded to have insulin resistance [17], [30]. For patients starting current anti-retroviral therapy regimen after January 2005, serum lipid profiles at less than 3 months, 6∼9 months, 12∼15 months, 18∼21 months, 24∼27 months, and 30∼33 months were recorded.

Patients were solicited about their average amount of alcohol consumption per week. A drink was defined as one can, bottle, or glass of beer, a glass of wine, a shot of liquor, a mixed drink with that amount of liquor, or any other kind of alcoholic beverage [31]. A patient who consumed more than seven drinks per week was considered to be a hazardous alcohol drinker [31], [32].

Human DNA was extracted using a kit (Geneaid Genomic DNA Mini Kit) according to the manufacturer’s instructions. Pro12Ala and C1431T polymorphisms of PPARγ and −803GA polymorphism of RBP4 were examined by real-time quantitative PCR (Applied Biosystems) using TaqMan Pre-Designed SNP Genotyping Assays.

Statistical analysis was performed with statistical software (SPSS, version 13.0). Continuous data were expressed as means ± standard deviations. The χ^2^ test with Yates’ correction or Fisher’s exact test was used for comparing categorical variables and the independent *t*-test was used for comparing continuous variables between two groups. A two-tailed *P* value less than 0.05 was considered to be statistically significant. Multivariate analysis was performed by binary logistic regression model. Variables of multivariate analysis include gender, age, C1430T polymorphism, P12A polymorphism, RBP4 polymorphism, hazardous drinking, HCV co-infection, calorie over-TEE, and efavirenz use, which have been shown to influence metabolic syndrome in HIV-infected patients in previous studies [25], [32], [33], [34]. The deviation from Hardy-Weinberg equilibrium for genotypes was tested by the χ^2^ test. The mixed effect model was used to examine the difference in serum triglyceride and cholesterol between patients with different gene variants over time. Bonferroni correction for multiple testing was applied. We performed post hoc analysis and statistical power test to exam the differences between pairs of groups after the global analysis.

## Results

There were 312 patients who had received two NRTIs plus efavirenz (a non-nucleoside reverse transcriptase inhibitor (NNRTI)) or lopinavir/ritonavir (a combination of protease inhibitor (PI)) at the National Cheng Kung University Hospital between Oct. 2000 and Jul. 2008. Among these patients, 114 patients fulfilling the inclusion criteria were evaluated. However, 23 patients declined to participate in the study resulting in 91 patients joining the study and their blood samples were collected for genetic polymorphism analyses. Of these 91 patients, 82 (90.1%) were males with a mean age of 44.4 years (Table 1). Mean duration of HIV infection was 70.2 months. Main risk factors for HIV infection were for men having sex with men in 6 (6.6%) patients and intravenous drug use in 33 (36.3%). The others were heterosexual or unknown. Mean duration of HAART was 40.6 months. Mean CD4^+^ cell count was 520.7 cells/mm^3^. As for HAART, 43 (47.3%) patients were treated by efavirenz plus two NRTIs and 48 (52.7%) by lopinavir/ritonavir plus two NRTIs. Patients with efavirenz therapy, than those with lopinavir/ritonavir-based regimens, had higher serum levels of fasting glucose (106.1 *vs*. 90.7 mg/dl, *P* = 0.01) and LDL (124.6 *vs*. 104.1 mg/dl, *P*<0.01), HOMA index (2.6 *vs*. 1.7, *P* = 0.02), but lower serum levels of uric acid (5.6 *vs*. 6.2 mg/dl, *P* = 0.03), and more often had hypercholesterolemia (cholesterol >200 mg/dl; 67.4 *vs*. 37.5%, *P* = 0.01) and serum LDL>110 mg/dl (72.1 *vs*. 41.7%, *P* = 0.01) (Table 2).

**Table 1:** Baseline clinical characteristics of 91 HIV-infected patients receiving lopinavir/ritonavir - or efavirenz-based antiretroviral therapy.

Clinical characters		TotalN = 91	Lopinavir/ritonavirN = 48		EfavirenzN = 43		*P*value		
Male gender		82 (90.1)	42 (87.5)		40 (93.0)		0.49		
Age, years		44.4±13.1	43.1±13.0		45.9±13.3		0.31		
Hazardous drinking		8 (8.8)	4 (8.3)		4 (9.3)		1.00		
Smoking		38 (41.8)	22 (45.8)		16 (37.2)		0.54		
Family history of DM		34 (37.4)	16 (33.3)		18 (41.9)		0.53		
Risk factor of HIV infectionIntravenous drug abuseMen having sex with men		6 (6.6)33 (36.3)	4 (8.4)20 (41.7)		2 (4.7)13 (30.2)		0.36		
Recognized duration of HIV infection, months		70.2±49.1	79.3±55.3		59.7±38.6		0.06		
Duration of antiretroviral therapy, months		40.6±25.9	35.2±23.3		46.7±27.6		**0.03**		
CD4^+^ count at enrollment, cells/mm^3^		520.7±226.1	516.8±220.5		525.1±235.0		0.86		
Daily calorie intake, kcal (88*)	1625.8±412.9			1645.7±396.7		1755.5±462.0		0.24	
Intake over-TEE, %		30 (33.0)	15 (31.9)		15 (36.6)		0.76		
Fat composition, %		36.1±9.7	36.3±9.3		36.0±10.2		0.94		
Body mass index (BMI), kg/m^2^BMI>24 kg/m^2^		22.8±3.431 (34.1)	22.8±3.517 (35.4)		22.9±3.414 (32.6)		0.880.95		
Waist circumference, cmWaist >90 (men) or >80 cm (women)Ratio of waist/hip circumference		78.9±9.711 (12.1)0.90±0.04	79.1±7.58 (16.7)0.90±0.04		78.8±11.83 (7.0)0.90±0.04		0.900.270.89		
Uric acid, mg/dl		5.9±1.3	6.2±1.3		5.6±1.3		**0.03**		
ALT, IU/ml		32.9±29.8	25.2±16.0		41.3±38.2		**0.01**		

Data are expressed as numbers (percentages) or mean values ± standard deviations.

TEE = total energy expenditure; ALT = alanine transaminase.

* The number of patients with indicated information.

**Table 2:** Genetic and metabolic characteristics of 91 HIV-infected patients receiving lopinavir/ritonavir- or efavirenz-based antiretroviral therapy.

Characters	TotalN = 91	Lopinavir/ritonavirN = 48	EfavirenzN = 43	*P*value
C1431T polymorphism, TC+TT genotype	44 (48.4)	23 (47.9)	21 (48.8)	1.00
P12A polymorphism, *Pro/Ala* genotype	8 (8.8)	4 (8.3)	4 (9.3)	1.00
RBP4 polymorphism, GA+AA genotype	21 (23.1)	11 (22.9)	10 (23.3)	1.00
Receipt of anti-hypoglycemic drugs	4 (4.4)	1 (2.1)	3 (7.0)	0.34
Fasting glucose, mg/dl	98.0±24.5	**90.7±9.0**	**106.1±32.7**	**0.01**
Fasting insulin, IU/ml	8.9±7.4	8.0±6.4	9.9±8.4	0.21
HOMA index	2.2±1.9	**1.7±1.3**	**2.6±2.2**	**0.02**
HOMA index >3.8	13 (14.3)	4 (8.5)	9 (20.9)	0.17
Receipt of anti-dyslipidemic drugs	35 (38.5)	18 (37.5)	17 (39.5)	1.00
Cholesterol, mg/dlCholesterol >200 mg/dl	205.9±42.647 (51.6)	198.3±49.3**18 (37.5)**	214.2±41.5**29 (67.4)**	0.10**0.01**
LDL cholesterol, mg/dlLDL cholesterol >110 mg/dl	113.8±34.151 (56.0)	**104.1±36.5** **20 (41.7)**	**124.6±27.7** **31 (72.1)**	**<0.01** **0.01**
HDL cholesterol, mg/dlHDL<40 (men) or <50 mg/dl (women)	45.4±11.727 (29.7)	44.7±12.217 (35.4)	46.1±11.110 (23.3)	0.570.30
Cholesterol/HDL ratio	4.9±1.9	4.8±2.3	4.9±1.5	0.94
Triglyceride, mg/dlTriglyceride >150 mg/dl	327.1±359.469 (75.8)	379.4±454.238 (79.2)	268.7±198.531 (72.1)	0.130.59

LDL = low-density lipoprotein; HDL = high-density lipoprotein.

**Figure 1:**
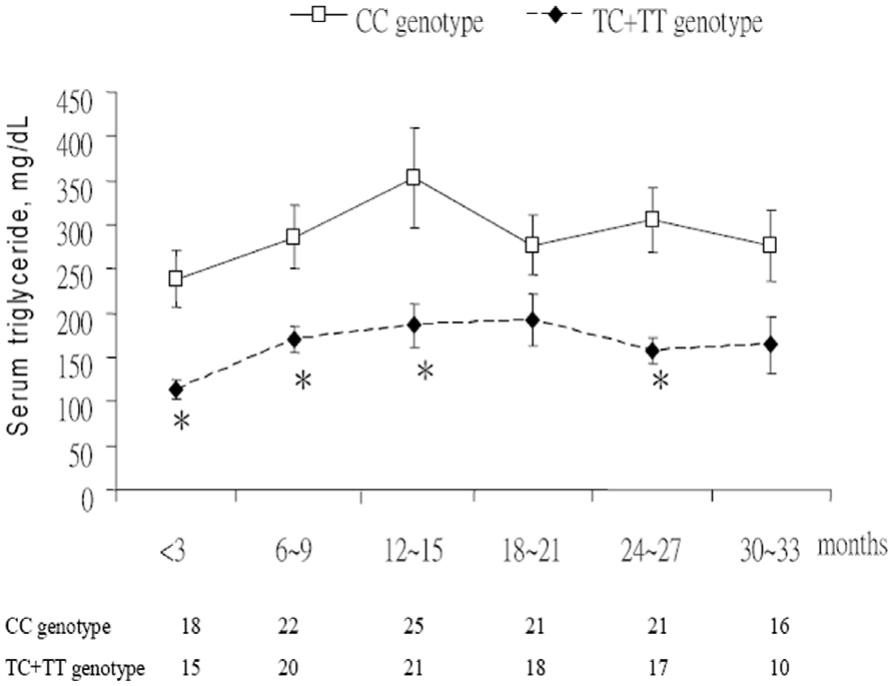
The influence of PPARγ C1431T polymorphism on serum triglyceride levels in HIV-infected patients after anti-retroviral therapy. The numbers below the months are the patient numbers. *P<0.05 for comparing serum triglyceride levels between patients with and without the T allele. *P* = 0.006 for difference in serum triglyceride levels over time in the mixed effect model.

For the C1431T polymorphism in PPARγ, 47 (51.6%) patients were the CC genotype, 41 (45.1%) CT genotype, and 3 (3.3%) TT genotype. Allele frequency for the C allele was 0.74 and T allele 0.26. The *P* value of χ^2^ test was 0.09 and such a result was consistent with the Hardy-Weinberg equilibrium. There was no discernible difference in the prevalence of smoking, hazardous drinking, presumed routes of HIV infection, HCV co-infection, duration of HIV infection, CD4^+^ cell counts, or HAART regimen (NNRTI or PI use) between patients with the T allele (CT+TT genotype) and without the T allele (CC genotype)(data not shown). No difference in BMI, waist circumference, systolic and diastolic blood pressure, fasting glucose and insulin, HOMA index, serum cholesterol, LDL, HDL and anti-dyslipidemic therapy was detectable between patients with and without the T allele (Table 3 and data not shown). Patients with the T allele had a trend toward lower rate of hypertriglyceridemia (triglyceride >150 mg/dl; 65.9 *vs*. 85.1%, *P* = 0.06; α = 0.05; statistical power = 0.57 in post hoc analysis) and had lower levels of serum uric acid (5.5 *vs*. 6.3 mg/dl, *P* = 0.01) than those without the T allele. While the multivariate analysis supported the protective effect of the T allele against development of hypertriglyceridemia (odds ratio [OR] 0.282, 95% confidence interval [CI] 0.087∼0.921, *P* = 0.04) (Table 4), there was no statistical significance under Bonferroni correction for multiple testing. For 46 patients with current anti-retroviral therapy after January 2005, their longitudinal lipid profiles were recorded. Serum triglyceride levels in patients with the T allele were significantly lower than those without the T allele at several time points after antiretroviral therapy (Figure 1). The effect is of statistical significance in serum triglyceride in patients with the T allele over time using the mixed effect model (*P* = 0.006, statistical power = 0.79). Although the differences of fasting insulin and HOMA index between patients with and without the T allele did not reach statistical significance, there were trends toward a lower fasting insulin level (7.5 *vs*.10.3 mg/dl; *P* = 0.07) and less insulin resistance (HOMA index >3.8; 6.8 *vs*. 21.7%; *P* = 0.09) in those with the T allele.

**Table 3:** PPARγ (C1431T and Pro12Ala) and RBP4 (−803GA) polymorphisms and metabolic syndrome among 91 HIV-infected patients.

Characters	PPARγ						RBP4			
	C1431T (rs3856806)			Pro12Ala (rs1801282)			−803GA (rs3758539)			
	CCN = 47(51.6%)	TC+TTN = 44(48.4%)	*P*value	*Pro/Pro*N = 83(91.2%)	*Pro/Ala*N = 8(8.8%)	*P*value	GGN = 70(76.9%)	GA+AAN = 21(23.1%)	*P*value	
Fasting glucose, mg/dl	95.0±16.5	101.2±30.7	0.23	98.1±25.3	96.4±15.1	0.85	95.0±11.2	107.8±46.2	0.22	
Fasting insulin, IU/ml	10.3±9.3	7.5±4.6	0.07	8.9±7.8	8.6±2.1	0.90	8.5±7.2	10.3±8.1	0.31	
HOMA index	2.4±2.1	2.0±1.6	0.29	2.2±1.9	2.1±0.8	0.89	2.0±1.6	2.8±2.5	0.15	
HOMA index >3.8	10 (21.7)	3 (6.8)	0.09	13 (15.9)	0 (0)	0.60	**6 (8.7)**	**7 (33.3)**	**0.01**	
Receipt of anti-hyperglycemic drugs	1 (2.1)	3 (6.8)	0.35	4 (4.8)	0 (0)	1.00	**1 (1.4)**	**3 (14.3)**	**0.04**	
Cholesterol, mg/dlCholesterol >200 mg/dl	206.7±44.823 (48.9)	204.9±48.324 (54.5)	0.850.75	204.2±47.340 (48.2)	223.1±30.37 (87.5)	0.270.06	206.3±46.638 (54.3)	204.3±46.09 (42.9)	0.870.50	
LDL cholesterol, mg/dlLDL cholesterol >110 mg/dl	113.9±34.427 (57.4)	113.7±34.124 (54.5)	0.980.95	**111.5±34.4**44 (53.0)	**138.0±18.5**7 (87.5)	**0.04**0.07	111.9±34.739 (55.7)	120.2±31.712 (57.1)	0.331.00	
HDL cholesterol, mg/dlHDL<40 (men) or <50 mg/dl (women)	44.7±11.413 (27.7)	46.1±12.114 (31.8)	0.570.84	45.3±11.725 (30.1)	46.3±11.82 (25.0)	0.831.00	46.0±12.121 (30.0)	43.4±10.06 (28.6)	0.391.00	
Triglyceride, mg/dlTriglyceride >150 mg/dl	355.9±421.140 (85.1)	296.3±280.729 (65.9)	0.430.06	335.2±374.163 (75.9)	243.3±113.46 (75.0)	0.491.00	335.8±400.051 (72.9)	298.1±168.318 (85.7)	0.680.36	
Receipt of anti-dyslipidemic drugs	16 (34.0)	19 (43.2)	0.50	31 (37.3)	4 (50.0)	0.48	26 (37.1)	9 (42.9)	0.83	
Uric acid, mg/dl	**6.3±1.3**	**5.5±1.3**	**0.01**	5.9±1.4	6.3±1.0	0.46	6.0±1.4	5.6±1.2	0.19	

Anti-hyperglycemic or anti-dyslipidemic medications use is expressed as number (percentage within each group); others are expressed as means ± standard deviations.

**Figure 2:**
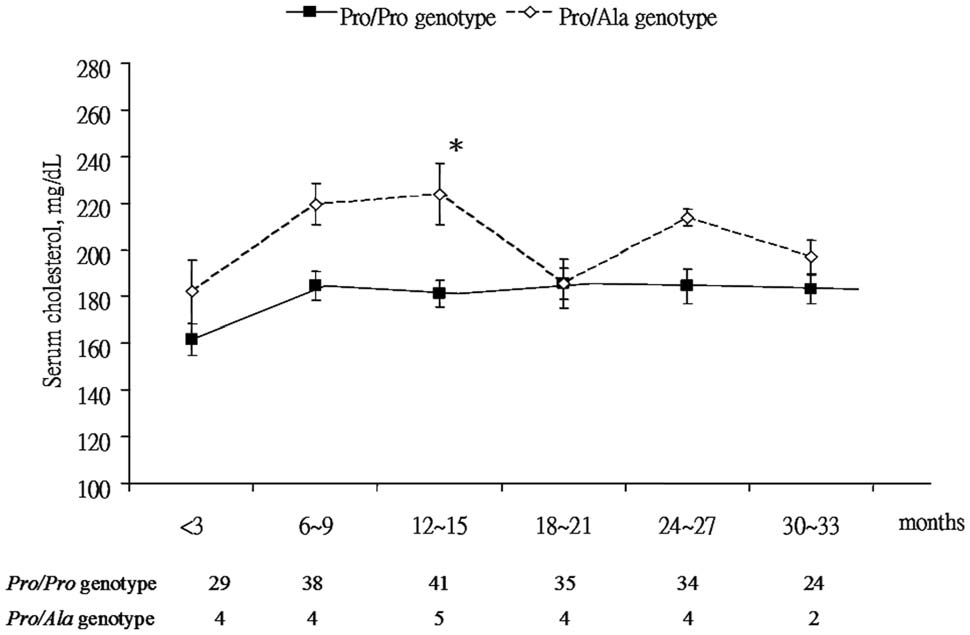
The influence of PPARγ Pro12Ala polymorphism on serum cholesterol levels in HIV-infected patients after anti-retroviral therapy. The numbers below the months are the patient numbers. *P<0.05 for comparing serum cholesterol levels between patients with and without the *Ala* allele. *P* = 0.04 for difference in serum cholesterol levels over time in the mixed effect model.

For the Pro12Ala polymorphism in PPARγ, 83 (91.2%) patients belong to the *Pro/Pro* genotype, and 8 (8.8%) the *Pro/Ala* genotype. No patient with the *Ala/Ala* genotype was identified. Allele frequency for the *Pro* allele was 0.96 and *Ala* allele 0.04. The *P* value of χ^2^ test for the Hardy-Weinberg equilibrium was 0.66. There was no difference in the prevalence of smoking, hazardous drinking, risk factors of HIV infection, HCV co-infection, duration of HIV infection, CD4^+^ cell counts, or HAART regimen between patients with the *Pro/Pro* genotype and with the *Pro/Ala* genotype (data not shown). For the metabolic profiles, such as BMI, waist circumference, blood pressure, fasting glucose and insulin, serum triglyceride, uric acid, and HDL, no difference was noted between two genotypes (Table 3 and data not shown). However, patients with the *Pro/Ala* genotype had a higher level of serum LDL (138.0 *vs*. 111.5 mg/dl, *P* = 0.04), and trends toward higher rates of hypercholesterolemia (87.5 *vs*. 48.2%, *P* = 0.06) and serum LDL>110 mg/dl (87.5 *vs*. 53.0%, *P* = 0.07) than those with the *Pro/Pro* genotype. The variables of hypercholesterolemia (OR: 7.373, 95% CI: 0.667∼81.524, *P* = 0.10) and serum LDL>110 mg/dl (OR: 6.946, 95% CI: 0.623∼77.481, *P* = 0.12) were found to be independent of the *Pro/Ala* genotype in the multivariate analysis (Table 4 and data not shown). Longitudinal profiles of serum cholesterol in 46 patients with antiretroviral therapy showed significant higher serum cholesterol levels in patients with the *Pro/Ala* genotype than those with the *Pro/Pro* genotype at 12∼15 months, and trends toward higher serum cholesterol levels at 6∼9 and 24∼27 months (Figure 2). There was marginal statistical significance in serum cholesterol in patients with the *Pro/Ala* genotype over time using the mixed effect model (*P* = 0.04, statistical power = 0.54).

**Table 4:** Multivariate analysis of factors associated with serum triglyceride >150 mg/dl, serum cholesterol >200 mg/dl, or HOMA index >3.8.

Genetic polymorphisms	triglyceride >150 mg/dl			cholesterol >200 mg/dl			HOMA index >3.8		
	Odds ratio	95% CI	*P* value	Odds ratio	95% CI	*P* value	Odds ratio	95% CI	*P* value
PPARγ C1430T (TC+TT)	0.282	0.087∼0.921	**0.04**	1.360	0.472∼3.920	0.57	0.272	0.049∼1.517	0.14
PPARγ Pro12Ala (*Pro/Ala*)	0.539	0.081∼3.583	0.52	7.373	0.667∼81.524	0.10	0.751	0.112∼5.063	0.77
RBP4 −803GA (GA+AA)	1.934	0.373∼10.027	0.43	0.943	0.255∼3.491	0.93	8.788	1.573∼49.087	**0.01**

Variables of multivariate analysis include gender, age, C1430T polymorphism, P12A polymorphism, RBP4 polymorphism, hazardous drinking, HCV co-infection, calorie over-TEE, and efavirenz use. CI denotes confidence interval.

For the −803GA polymorphism in RBP4, 70 (76.9%) patients belong to the GG genotype, 19 (20.9%) GA genotype, and 2 (2.2%) AA genotype. Allele frequency for the G allele was 0.87 and A allele 0.13. The *P* value of χ^2^ test for the Hardy-Weinberg equilibrium was 0.60. Patients with the A allele (GA+AA genotype) more often had insulin resistance (33.3 *vs*. 8.7%, *P* = 0.01; α = 0.05; statistical power = 0.72 in post hoc analysis) and more often had treated for DM (14.3 *vs*. 1.4%, *P* = 0.04) than those without the A allele (GG genotype) (Table 3). Furthermore, the multivariate analysis supported the A allele as a predictor of insulin resistance with an odds ratio of 8.8 (95% CI, 1.6∼49.1, *P* = 0.01) (Table 4). It is of statistical significance under Bonferroni correction for multiple testing (*P*<0.017).

## Discussion

In this study, we investigated the effect of PPARγ and RBP4 polymorphisms on body mass, insulin resistance and dyslipidemia among HIV-infected patients with anti-retroviral therapy (HAART). Our results showed that the T allele of PPARγ C1431T polymorphism was marginally associated with a lower rate of hypertriglyceridemia in HIV-infected patients receiving anti-retroviral therapy. However, the borderline significance together with insignificant power did not support the protective effect of the T allele against development of hypertriglyceridemia. No dramatic impact of the *Ala* allele of PPARγ Pro12Ala polymorphism on the metabolic parameters we examined was identified. Finally, the A allele of RBP4 −803GA polymorphism was associated with a higher rate of insulin resistance in HIV-infected patients receiving anti-retroviral therapy.

HIV-infected patients with lipodystrophy had been found to have a lower PPARγ level in the subcutaneous adipose tissue, and a higher waist-to-hip ratio, higher serum insulin and triglyceride levels [17]. Although genetic variants of PPARγ have been associated with insulin sensitivity and glucose metabolism in non HIV-infected people, the influence of PPARγ polymorphism on the metabolism in HIV-infected patients receiving HAART was not conclusive in previous studies [13], [14], [35]. The inconsistency of PPARγ genetic variation on the metabolism can stem from the influences of diet, drinking, or antiretroviral therapy in each HIV-infected individual. For example, increased fat intake is associated with a higher serum triglyceride level in HIV-infected patients [25], [36]. Similarly, hazardous alcohol consumption is associated with several metabolic consequences in HIV-infected patients, such as lipodystrophy and the increases in cholesterol, triglyceride and LDL [32], [33]. To our knowledge, our study is the first one to assess the influence of genetic factor of PPARγ on metabolism after multivariate analyses adjusting for different factors, such as diet, drinking, and antiretroviral regimens, on BMI, waist circumference, insulin resistance and dyslipidemia of HIV-infected patients.

Although C1431T is a silent polymorphism, it was found to be a better predictor of fasting insulin levels and insulin sensitivity than Pro12Ala, suggesting that the C1431T polymorphism may be in tight linkage disequilibrium with a functional variant in PPARγ or nearby gene [37], [38]. While, in non HIV-infected patients, the effects of PPARγ C1431T polymorphism on metabolic syndrome varied in different studies, the majority of them indicated the association of C1431T polymorphism with obesity and hyperglycemia. For example, in a Finnish study, obese women with the TT genotype had an increased BMI and waist circumference [39]. Similarly, in Chinese population, the CT and TT genotypes in C1431T polymorphism have been associated with a higher fasting blood sugar in patients with metabolic syndrome [35]. However, in an Asian population, the CT and TT genotypes had a lower risk of diabetes, but a higher BMI than those with the CC genotype [14]. In HIV-infected patients, the association of C1431T polymorphism with metabolic syndrome is not well documented. For example, Nazih *et al.* found there was no convincing association between the His449His (equal to C1431T) polymorphism and individual components of the metabolic syndrome [40]. In the study by Zanone *et al.*, the C161T (equal to C1431T) polymorphism had no influence on the presence of atrophy and fat accumulation in individuals with HIV-related lipodystrophy [41]. In our study, we found that the T allele of PPARγ C1431T polymorphism was marginally associated with a lower rate of hypertriglyceridemia in HIV-infected patients receiving antiretroviral therapy in univariate and multivariate analyses. However, it did not reach a desirable power in the post hoc analysis and statistical significance after the correction for multiple testing. Finally, the statistical significance (0.006) with a nearly acceptable power (0.79) indicates that the T allele carriers have lower serum triglyceride levels at several time points of the longitudinal follow-up. There are many reasons to reduce the power of mixed models. For example: smaller sample size, lower number of repeated measures, higher between-subject variance, higher intraclass correlation, and smaller difference between two groups, and so on. Thus, the effect of PPARγ C1431T polymorphism on serum triglyceride in HIV-infected patients is not significant and requires further large scale study.

The Pro12Ala polymorphism in PPARγ represents the first genetic variant with a broad impact on the risk and complications of type 2 diabetes. *In vitro* studies showed that *Ala* variant exhibited moderate reduction of target gene transactivation due to decreased DNA binding capacity [13]. The effect of the *Ala* carriage is not conclusive in the literature both in non HIV-infected and in HIV-infected patients. While most studies in non HIV-infected patients found that the *Ala* carrier exerted a protective effect from development of type 2 diabetes, insulin resistance or obesity, some studies found it to have a deleterious effect [14], [42], [43], [44]. In Chinese population, the *Ala* carrier was associated with a higher fasting level of blood sugar in patients with the metabolic syndrome [35]. Similarly, the influence of the *Ala* carriage on metabolism in HIV-infected patients is not evident. For example, in the study by Saumoy *et al.*, the Pro12Ala polymorphism had no effect on the risk of developing lipodystrophy in HIV-1-infected patients treated with HAART [45]. In the study by Nazih *et al.*, there was no convincing association between the Pro12Ala polymorphism and individual components of the metabolic syndrome, except for the association of the Pro12Ala polymorphism with diabetes in HIV-infected patients [40]. Thus, these suggest that Pro12Ala polymorphism is unlikely to have a significant impact on the metabolic syndrome in HIV-infected patients. Consistently, our study showed that the *Ala* carriers of Pro12Ala polymorphism in PPARγ were not associated with serum levels of cholesterol and LDL in HIV-infected patients. While the *Ala* carriers had higher serum cholesterol levels at several time points of the longitudinal follow-up, the borderline significance (0.04) together with insignificant power (0.54) makes the results inapplicable at a population level. We noticed relatively few *Ala* carriers in our study, which may be related to the insignificant impact of *Ala* carriage on lipid metabolism in our findings. Thus further studies with more patients are warranted to reveal the impact of Pro12Ala polymorphism on metabolism in HIV-infected patients.

Because the −803GA polymorphism is located in the enhancer region, the G>A substitution alters transcriptional efficiency and affinity of the enhancer sequence for the transcription factor HNF1α [46]. The A allele of RBP4 −803GA polymorphism was linked to an increased risk of type 2 diabetes in non HIV-infected population [21]. In HIV-infected patients receiving HAART, the serum RBP4 level has been positively correlated with obesity, insulin resistance and dyslipidemia [24]. However, the role of the A allele of RBP4 −803GA polymorphism in HIV-infected patients is still unknown. Our study revealed that the A allele of RBP4 −803GA polymorphism was associated with insulin resistance in HIV-infected patients receiving HAART. This provides us another potential surrogate marker of insulin resistance in HIV-infected patients receiving antiretroviral therapy.

It has long been noted that PI treatment was associated with dyslipidemia, particularly hypertriglyceridemia [47]. The class of NNRTI, in contrast, induces dyslipidemia to a lesser degree than PI. However, NNRTI-related dyslipidemia can still be found in several studies [48], [49], [50]. The effect of NNRTI or PI therapy on the lipid profile of HIV-infected patients in Asia is not clear. In our study, patients with efavirenz therapy had higher serum levels of fasting glucose, LDL, and HOMA index, lower serum levels of uric acid, and more often had hypercholesterolemia and LDL>110 mg/dl, than those with lopinavir/ritonavir-based regimens. These results suggest that efavirenz may be associated with more metabolic side effects than lopinavir/ritonavir in Asian population. Thus, further randomized, double-blind trials will be required to clarify the effect of efavirenz or lopinavir/ritonavir on the metabolic profile in an Asian population.

There are several limitations in our study. First, the case number is relatively small in our study. Many effects of exposure may not exhibit a logistic difference and dose-response effect in our study. Second, exercise habits, which may influence BMI, insulin resistance or lipid profiles of our patients, were not recorded. Third, our study is primarily a cross-sectional study, and some longitudinal effects may not be observed in this setting. Fourth, we did not measure serum levels of RBP4 in these patients due to no permission from the Institutional Review Board and no available stored serum samples. However, it is the first study revealing the different effects of PPARγ and RBP4 polymorphisms on the metabolic syndrome after multivariate analysis adjusting for anti-retroviral drug, diet and drinking in HIV-infected patients receiving anti-retroviral therapy.

In conclusion, the A allele of −803GA polymorphism in RBP4 is associated with a higher rate of insulin resistance. These results suggest that certain genetic factors can affect the metabolic syndrome in HIV-infected patients receiving anti-retroviral therapy. Identification of the individuals with unfavorable genotypes may be helpful to select more appropriate drugs to minimize the risk of metabolic syndrome. Moreover, the knowledge of function alterations of the vulnerable genes could be used as the surrogates of therapeutic targets in the future.
